# Cell therapy of periodontium: from animal to human?

**DOI:** 10.3389/fphys.2013.00325

**Published:** 2013-11-15

**Authors:** Elena A. Trofin, Paul Monsarrat, Philippe Kémoun

**Affiliations:** ^1^Department of Biology, Toulouse Faculty of Dentistry - Paul Sabatier University, Toulouse University Hospital - CHU de ToulouseToulouse, France; ^2^Department of Pathology, Faculty of Medicine and Odontology, University of ValenciaValencia, Spain; ^3^Department of Public Health, Toulouse Faculty of Dentistry - Paul Sabatier University, Toulouse University Hospital - CHU de ToulouseToulouse, France

**Keywords:** mesenchymal stromal cells, periodontitis, tissue engineering, bone regeneration, clinical trials as topic

## Abstract

Periodontitis is a chronic inflammatory disease affecting the soft and hard tissues supporting the teeth, which often leads to tooth loss. Its significant impact on the patient's general health and quality of life point to a need for more effective management of this condition. Existing treatments include scaling/root planning and surgical approaches but their overall effects are relatively modest and restricted in application. The goal of regenerative therapy of periodontal defects is to enhance endogenous progenitors and thus promote optimal wound healing. Considering that the host or tissue might be defective in the periodontitis context, it has been proposed that grafting exogenous stem cells would produce new tissues and create a suitable microenvironment for tissue regeneration. Thus, cell therapy of periodontium has been assessed in many animal models and promising results have been reported. However, the methodological diversity of these studies makes the conversion to clinical practice difficult. The aim of this review is to highlight the primary requirements to be satisfied before the leap to clinical trials can be made. We therefore review cell therapy applications for periodontal regeneration in animal models and the concerns to be addressed before undertaking human experiments.

## Introduction

Periodontitis is an immuno-infectious disease, characterized by loss of both the soft and hard tissues anchoring the teeth. Left untreated, it leads to tooth loss (Pihlstrom et al., 2005). Chronic periodontitis is found in 15–50% of adults in developed countries (Oliver et al., 1998; Bourgeois et al., 2005). Conventional treatment, including oral hygiene instructions, and scaling/root planing, aims to prevent the disease, or slow or stop its progress, and maintain the therapeutic goals achieved but is usually insufficient to promote the regeneration of damaged structures (Bosshardt and Sculean, 2009). Deep infrabony defects associated with periodontal pockets are the classic indication for surgical periodontal regenerative therapy (Ramseier et al., 2012). Nevertheless, outcomes of existing procedures [guided tissue regeneration (GTR), Enamel-Matrix Derivatives (EMD) or Platelet Rich Plasma (PRP)], are not predictable and are associated with a relatively high degree of variability (Needleman et al., 2006; Esposito et al., 2009).

The regenerative mechanisms of mesenchymal stromal cell (MSC) grafts include direct commitment toward differentiating cells together with paracrine communication with resident connective cells, and infiltration of inflammatory cells, antigen-presenting cells, or both (Sorrell and Caplan, 2010). Paracrine interactions require these cells to produce and respond to a variety of trophic factors that may stimulate the resident cells to differentiate and themselves renew the pathological tissue (Baraniak and McDevitt, 2010). In addition, MSCs also exhibit immuno-modulatory functions, making them a potential tool to combat an immuno-infectious disease such as periodontitis. The reduction of inflammation by MSCs may halt the development of injury and allow regenerative processes to take place (Sensebe et al., 2010). Furthermore, MSCs may exert a neovascularization effect (Wu et al., 2007). Thus, one of the therapeutic functions of MSCs is the early induction of granulation tissue followed by the stabilization of the neovascular network in the periodontal niche (Sorrell and Caplan, 2010).

The graft of exogenous cells producing new tissues and/or making the local microenvironment suitable for an optimal activation of *in-situ* progenitors (Ilic and Polak, 2012; Shin and Peterson, 2013) has been tested in many models. Adult multipotent mesenchymal stromal cells, from bone marrow (BMSCs) or adipose tissue (adipose-derived stem cells or ASCs), are promising in the treatment of human diseases like graft-vs-host disease, ischemic cardiovascular diseases or large bone defects (Bourin et al., 2010; Sensebe et al., 2010). The main features of such cells include a capacity to self-renew and to undergo extensive proliferation and differentiation to various mesenchymal lineages (Dominici et al., 2006). Considering that the host or tissue might be defective in the periodontitis context, grafting exogenous MSCs that produce new tissues and create a suitable microenvironment for tissue regeneration has been proposed (Chen et al., 2012a). However, the methodological diversity of the available studies makes the conversion to clinical practice difficult. The aim of this review is, first, to report recent findings of periodontal regeneration in animal models and, second, to highlight the primary requirements to be satisfied before the leap to clinical trials can be made.

## Results from animal studies

Advances in mesenchymal stem cell isolation, growth factor biology, and biodegradable polymer constructs have set the stage for successful tissue engineering of the periodontium, basically in animal models. Our screening of the literature revealed that data were available for about fifty studies. The majority of them concerned periodontal defects generated mechanically (burs) on dogs and rodents. Except for nude rodents where xenogeneic cells were used, most studies used autologous cell grafts. Periodontal ligament stromal cells (PDLSCs) were employed in about half the studies.

Table 1 outlines the general methodology from recent papers investigating periodontal regeneration of various defect models in a range of large animals. These studies were selected both for their methodological quality and to reflect the heterogeneity of methodologies used. We voluntarily did not select studies on rodents because of the low similarity between their physiopathology and that of humans, although they are considered as essential models for preliminary protocols before moving on to large-animal trials. Overall, the outcomes reported (Table 2) suggest that MSCs have the ability to enhance the regeneration of functional periodontal apparatus: newly formed bone and cementum with well-oriented ligament fibers.

**Table 1:** **General methodology of cell therapy in animal subjects**.

**References**	**Animal**	**Type of defect**	**Cell type**	**Carrier**	**Groups**	**Outcomes**
			**Type of graft**			
Akizuki et al., *J. Periodontal. Res.*, 2005	Dog	Fenestration defects	PDLSCs Autologous	PDLSC single-layered sheets, reinforced using hyaluronic acid sheet and applied onto roots	PDLSC sheet with hyaluronic acid carrier Hyaluronic acid alone	Histomorphometric Post-operative complications
Chen et al., *Gene Ther.*, 2008	Rabbit	Fenestration defects Trans gingival periodontal defect	BMSCs transfected to over-express BMP-2 Autologous	Pluronic F127, solidified into gel form in incubator	BMP-2 gene-transfected BMSCs with PF127 Control transfected BMSCs with PF127 Untransfected BMSCs with PF127 PF127 only	Histologic 3D micro-CT Ankylosis Post-operative complications
Ding et al., *Stem Cells*, 2010	Minipig	Three-wall infra-bony defects	PDLSCs Autologous Allogeneic	PDLSCs cultured with HA/TCP to obtain cell sheets. Gelatin membranes covered defects	Autologous cells with HA/TCP Allogeneic cells with HA/TCP Autologous heterogenic with HA/TCP HA/TCP only No carrier	Clinical assessments Histomorphometric Immune assays Rejection
Fawzy El-Sayed et al., *J. Clin. Periodontol.*, 2012	Minipig	Two-wall infra-bony defects Silk sutures around cervical region of teeth were used to induce inflammation	Gingival margin-derived progenitor cells Autologous	Inorganic: deproteinized bovine cancellous bone (DBCB) Organic: collagen scaffold A collagen membrane was added to cover defects	Cells with DBCB Cells with collagen scaffold DBCB only Collagen scaffold only No carrier	Clinical assessments Radiographic Histomorphometric Ankylosis Root resorption
Hasegawa et al., *J. Periodontol.*, 2006	Dog	Class III furcation defect	BMSCs Autologous	Atelocollagen	BMSCs in atelocollagen Atelocollagen only	Histologic Immunohistochemistry
Iwata et al., *Biomaterials*, 2009	Dog	Three-wall infra-bony defect	PDLSCs Culture in an osteogenic medium Autologous	Tri-layered cell sheets formed with PDLSCs and sheets of polyglycolic acid (PGA). Defects were also filled with β TCP	Cell sheets with β TCP PGA sheets only with β TCP	Histomorphometric Ankylosis Post-operative complications
Li et al., *Cells Tissues Organs*, 2009	Dog	Fenestration defects	BMSCs, cryopreserved or not	Collagen membranes serving as carrier and applied onto roots. e-PTFE membranes covered defects	Cryopreserved BMSCs Non-cryopreserved BMSCs No cells	Histomorphometric Post-operative complications
Liu et al., *Stem Cells*, 2008	Minipig	Two-wall infra-bony defects Silk sutures around cervical region of teeth were used to induce inflammation	PDLSCs	PDLSCs combined with HA/TCP with a gelatin membrane covering	PDLSCs with HA/TCP HA/TCP only No treatment	Clinical observations Histomorphometric Radiographic
Nunez et al., *J. Periodontal. Res.*, 2012	Dog	Three-wall infra-bony defects	Cementum and periodontal ligament-derived cells (CDCs and PDLSCs) Autologous	Collagen sponge	Collagen sponge with PDLDCs Collagen sponge with CDCs Collagen sponge with culture medium (control)	Histomorphometric Clinical measurements Post-operative complications Ankylosis Root resorption
Park et al., *Cell Transplant.*, 2011	Dog	Fenestration defects: apical involvement defects	Dental pulp, periodontal ligament and peri-apical follicular stem cells Autologous	None	PDLSC graft group Dental pulp stem cell graft group Peri-apical follicular stem cell graft group	Clinical measurements 3D micro-CT Histologic Post-operative complications Neoplasm formation
					No surgical intervention Periodontal defect without cells	
Simsek et al., *Clin. Oral Investig.*, 2012	Dog	Class II furcation defect Defects were filled with rubber impression paste to induce inflammation	BMSCs Autologous	PRP mixed with BMSCs and autogenous cortical bone (ACB) also added into defects	BMSCs with PRP and ACB PRP and ACB PRP alone ACB alone No carrier (scaling and root planning only)	Clinical observations Histomorphometric Post-operative complications Root resorption Ankylosis
Suaid et al., *J. Clin. Periodontol.*, 2012	Dog	Class III furcation defect	PDLSCs Autologous	Collagen sponges were seeded with cells and membranes were used for guided tissue regeneration	Collagen sponge with cells Collagen sponge without cells Collagen sponge only Guided tissue regeneration only Surgical act only	Clinical observations Histomorphometric Post-operative complications Root resorption Ankylosis
Suaid et al., *J. Clin. Periodontol.*, 2011	Dog	Class II furcation defect	PDLSCs Autologous	Collagen sponges were seeded with cells and membranes were used for guided tissue regeneration	Collagen sponge with cells Collagen sponge without cells	Clinical observations Histomorphometric Post-operative complications Ankylosis
Takedachi et al., *J. Oral Biosci.*, 2013	Dog	Two-wall and furcation class II defects	ASCs Autologous	Fibrin gel	ASCs mixed with fibrin gel Fibrin gel alone	Radiographic 3D micro-CT Histologic Post-operative complications
Tobita et al., *Cytotherapy*, 2013	Dog	Class III furcation defect	ASCs Autologous	Autologous PRP was prepared to be mixed with cells	ASC seeded in PRP PRP alone No implantation	Radiographic Histomorphometric Immunohistochemistry
Tsumanuma et al., *Biomaterials*, 2011	Dog	One-wall infra-bony defect	Alveolar periosteum-derived stromal cells, PDLSCs and BMSCs Autologous	Tri-layered cell sheets were constructed using PGA, defects were also filled with β-TCP and type I collagen	Cell sheets with BMSCs Cell sheets with PDLSCs Cell sheets with APCs PGA sheet without cells	Clinical observations Histomorphometric Immunohistochemistry Post-operative complications Ankylosis Root resorption

Abbreviations: ACB, autogenous cortical bone; ASC, adipose-derived stem cell; BMP2, bone morphogenetic protein-2; BMSC, bone marrow stromal cell; DBCB, deproteinized bovine cancellous bone; CDC, cementum derived cell; e-PTFE, expanding polytetrafluoroethylene; HA, hydroxyapatite; PDLSC, periodontal ligament stromal cell; PF127, Pluronic F127; PGA, polyglycolic acid; TCP, tricalcium phosphate.

**Table 2:** **Results from animal studies**.

**References**	**Experimental group outcomes**	**Control group outcomes**	**Conclusion**
Akizuki et al., *J. Periodontal. Res.*, 2005	Periodontal tissue healing with bone, cementum, and periodontal ligament formation was observed in three defects. Signs of ankylosis were observed in some specimens.	No cementum was formed, only one defect showed new bone. Parallel connective tissue existed adjacent to the denuded root surface.	The periodontal ligament cell sheet applied in a dehiscence-type defect resulted in regeneration of periodontal tissues in beagle dogs.
Chen et al., *Gene Ther.*, 2008	BMP-2 gene-infected BMSCs Newly formed periodontal ligament fibers were functionally orientated and new connective tissue fibers had been inserted into both the new cementum and the new bone.	BMSCs Woven bone was formed from apical part of defects to middle of the roots. There were small resorption areas with new cementum and fibers.	Regeneration of the periodontal attachment apparatus was enhanced by cells engineered to express BMP-2 gene.
Ding et al., *Stem Cells*, 2010	Both the autologous and allogeneic PDLSC treatments significantly improved periodontal tissue regeneration compared with the HA/TCP and control groups. New bone, cementum, and periodontal ligament were regenerated to normal levels in both the autologous and allogeneic PDLSC groups.	Limited or partial periodontal tissue regeneration in the control groups and HA/TCP group. Little alveolar bone recovery.	A sheet of minipig PDLSCs can repair allogeneic bone defects in an experimental model of periodontitis.
Fawzy El-Sayed et al., *J. Clin. Periodontol.*, 2012	Higher clinical attachment level, probing depth and lower gingival recession. Thin multi-layered squamous sulcular epithelium. Regeneration of bone, cementum, and periodontal ligament with Sharpey's fibers similar to normal periodontium. Higher histological attachment level, lower junctional epithelium length, and connective tissue adhesion.	Thicker multi-layered squamous sulcular epithelium. Periodontal tissue loss, unorganized Sharpey's fibers, root resorption, and ankylosis.	Gingival margin-derived stem/progenitor cells show significant periodontal regenerative potential.
Hasegawa et al., *J. Periodontol.*, 2006	New cementum. New regenerated periodontal ligament separating the new bone from the cementum. No complete alveolar bone reconstruction.	Insufficient periodontal regeneration. Epithelial cells invading top of the furcation, and no cementum regeneration.	Transplanted BMSCs can survive and differentiate into periodontal tissue–composing cells, resulting in enhancement of periodontal tissue regeneration.
Iwata et al., *Biomaterials*, 2009	Complete bone filling with an appropriate space of periodontal ligament was observed. Complete periodontal regeneration with both newly formed bone and cementum connecting with well-oriented collagen fibers.	Almost 50% of bone filling was observed.	Transplantable multi-layered PDLSC cell sheets were successfully fabricated and induced a true periodontal system, including alveolar bone, cementum, and well-oriented fibers at the same time.
Li et al., *Cells Tissues Organs*, 2009	Both cryo- and non-cryopreserved BMSC groups exhibited periodontal regeneration. New PDL was formed between the new alveolar bone and cementum with Sharpey's fibers extending into the newly formed cementum and bone. Cementum and PDL were fully regenerated.	Very little regenerated alveolar bone and cementum. PDL fibers were parallel to the root surface. Small lacunae of resorption were present on roots.	Cryopreserved BMSCs showed no altered regenerative capacity compared with freshly isolated BMSCs in the application of periodontal regeneration.
Liu et al., *Stem Cells*, 2008	New bone and periodontal tissues were regenerated with newly formed Sharpey's fibers anchored into the newly regenerated cementum. Nevertheless, bone was not regenerated to normal level.	Fibers lacking the typical structure of Sharpey's fibers filled in the periodontal defect. Residual inflammation was still present.	The study demonstrated the utility of using an autologous PDLSC therapeutic approach to treat periodontitis in a miniature pig preclinical model.
Nunez et al., *J. Periodontal. Res.*, 2012	Histological characteristics of periodontal regeneration: formation of new cellular cementum, no signs of root resorption or ankylosis, rich capillary vessels. Greater new-bone formation in the PDLDC group.	Healing by repair, with limited formation of new cellular cementum. No signs of root resorption or ankylosis.	Cellular therapy, in combination with a collagen sponge, promoted periodontal regeneration in experimental infra-bony periodontal defects.
Park et al., *Cell Transplant.*, 2011	Healing response was favorable for all treatment groups. For PDLSC group, incremental lines of neocementum were observed, with Sharpey's fibers inserted and cellular cementum at the root apex.	No tissue attachment but presence of surrounding granulation.	PDLSCs may significantly promote periodontal regeneration in class II furcation defects in dogs. Authors suggested PDLSCs were the best candidates for regeneration.
Simsek et al., *Clin. Oral Investig.*, 2012	Formation of new cementum and coronal growth of alveolar bone were observed in all groups. No root resorption or ankylosis was present. No efficacy difference between the groups was found for alveolar bone formation. There was no severe inflammation or swelling and dehiscence of the flaps. Regeneration of cementum for cell group was significantly higher than control group.		Periodontal regeneration with complete filling of class II furcation defects with cementum, alveolar bone, and periodontal ligament was obtained for all groups compared to control group.
Suaid et al., *J. Clin. Periodontol.*, 2011	Woven bone was predominant. In all groups, new cementum and obliquely oriented periodontal fibers were regenerated. Ankylosis was present in one specimen for each group. Nevertheless, cell group presented more new cementum surface, less connective tissue and epithelium along root surface, more bone area than control group.	Large bone marrow spaces were predominant. Down-growth of epithelium was observed in some histological sections.	PDLSCs with guided tissue regeneration were shown to be efficient for periodontal regeneration in class II furcation defects.
Suaid et al., *J. Clin. Periodontol.*, 2012	Cell-treated group exhibited larger area of new bone, more cementum, and more periodontal regeneration than other groups. Complete filling of the furcation was achieved in 2 out of 6 defects.	All defects showed gingival recession with exposure of the furcation area. Defects were incompletely filled, with inflamed connective tissue covered by gingival epithelium. There was no cementum covering entire root area.	PDLSCs in association with guided tissue regeneration may significantly promote periodontal regeneration in class III furcation defects surgically created in dogs.
Takedachi et al., *J. Oral Biosci.*, 2013	Compared to control, bone mineral density increased in 2-wall defects. New bone and new cementum were formed, with connective tissue fibers inserted vertically in the furcation class II defect.		A mix of ASCs and fibrin gel promoted periodontal regeneration in beagle dogs.
Tobita et al., *Cytotherapy*, 2013	New bone was formed and periodontal complex was regenerated after 2 months. Osteocalcin-positive cells were found on the surface of the dentin.	Ingrowth of epithelium into the defect was found in the non-implanted group. Granular tissue and radiolucency were observed in both control groups.	Efficacy of the combination of ASCs and PRP in canine periodontal tissue regeneration.
Tsumanuma et al., *Biomaterials*, 2011	PDLSC group showed more cellular and acellular cementum than other groups. Dense collagen fibers were perpendicularly attached to the cementum-like tissue layer. In BMSC group, fibers were obliquely oriented whereas they were parallel in APC group.	Collagen fibers were sparse. Alveolar bone regeneration was observed in all groups.	PDLSC sheets combined with β-TCP and collagen induced more periodontal regeneration than in other groups.

Abbreviations: ASC, adipose-derived stem cell; BMP2, bone morphogenetic protein-2; BMSC, bone marrow stromal cell; HA, hydroxyapatite; PDL, periodontal ligament; PDLSC, periodontal ligament stromal cell; TCP, tricalcium phosphate.

## Results from human studies

We identified 7 case reports (for a total of 22 patients) where MSCs had been applied clinically for infra-bony defects and furcation involvement after chronic periodontitis in humans. The efficacy of human periodontal cell therapy by grafting autologous stromal cells from gingiva or periodontal ligament with a hydroxyapatite (HA) carrier has been assessed since 1992 (Feng and Hou, 1992; Feng et al., 1995, 2010; Hou et al., 2003). Cultured mandibular periosteum-derived cell sheets with PRP have also been used in the treatment of chronic periodontitis without (Mizuno et al., 2010) or with HA in patients suffering from advanced chronic periodontitis (Okuda et al., 2009). A BMSC-PRP gel has also been used in an infrabony periodontal defect and led to a 4 mm clinical attachment gain (Yamada et al., 2006). Results suggest promising improvements in pocket depth, attachment gain and tooth mobility, compared to control groups when present. These data suggest MSCs may induce efficient and safe periodontal regeneration in humans.

Through the International Clinical Trials Registry Platform (World Health Organization), we identified four clinical studies (last access 2013/08/27): two single-arm studies using (i) ASCs for 12 patients with deep infra-bony defects (recruiting) and (ii) a mixture of *ex-vivo* cultured MSCs and *ex-vivo* cultured osteoblast-like cells differentiated from MSCs for 10 patients with chronic periodontitis (completed); and two trials investigating the safety and efficacy of PDLSCs in chronic periodontitis, one randomized and one non-randomized controlled trial, with respectively 35 and 80 patients (recruiting).

Nevertheless, results from case reports and clinical trials should be interpreted with caution. Although the clinical potential of MSCs in tissue regeneration appears to be established, the mechanisms involved in these processes after transplantation are not clearly understood (Chen et al., 2012b; Hoogduijn and Dor, 2013). Moreover, the clinical indications in periodontology remain be defined (Chen and Jin, 2010); the multiplicity of factors to be taken into account makes the regeneration equation more complex. Future studies should discuss regeneration according to the type of periodontal defects (number of walls), the type of periodontitis (chronic, aggressive) or the method used to generate the periodontitis model in animals, whether the MSCs are autologous, allogeneic or xenogeneic, the type of animal (dog, pig), the source tissue of MSCs (dental, oral, or extra-oral sources), the scaffold used, adjunctive growth factors or specific culture medium, etc.

## Key elements to consider before making the step to clinical trials

The transfer of pre-clinical data to the clinical setting could be challenging and time consuming. So we will discuss some elements related to the choice of the origin of MSCs, their potential toxicity and their carrier, some laboratory considerations, the extent to which animal models reflect the applicability of the technique in humans, and regulatory information.

### Cell sources

One of the most important items of information that could help with the predictability of clinical results is the type of cell used. On the one hand, each tissue source has its own biological features and, even when they have common surface markers, these cells are determined by their original environment and may possibly be involved in specific differentiation pathways (Lin et al., 2009). On the other hand, the local microenvironment and surrounding tissue are important factors that influence the cell fate of whatever cells are ultimately used (Chen et al., 2011).

As stated above, PDLSCs are the cells most used for clinical trials, probably because of their periodontal origin and promising results in both animal and human trials (Feng et al., 2010; Suaid et al., 2012). Even if the choice of stromal cells from the oral niche (pulp, ligament, gingiva or oral alveolar bone) seems rational when the aim is to regenerate periodontal structures (Lin et al., 2009), periodontitis defects need a large number of cells (about 10^7^ cells are needed for one defect), which would be impossible to obtain from a single subject. Thus, the respect of ethical considerations (e.g., putative removal of healthy teeth, increasing genetic instability through passages during cellular expansion) requires other sources of MSCs to be sought.

Two major locations of available MSCs are long-bone marrow and adipose tissue. These cells are morphologically and immunophenotypically similar to PDLSCs (Huang et al., 2009). Unlike BMSCs, ASCs can be recovered easily in large numbers by means of liposuction under local anesthesia. Indeed, adipose tissue is the richest source of MSCs, 100 times more than bone marrow (Bourin et al., 2010). Although ASCs may exhibit a reduced ability to differentiate into bone and cartilage (Kern et al., 2006) compared to BMSC or oral mesenchymal cells, they have given promising results in periodontal regeneration in dogs (Takedachi et al., 2013; Tobita et al., 2013).

The need for high cell concentrations requires the optimal type of transplant to be determined. To become a clinical reality, xenografts need to overcome immunological, physiological, and infectious obstacles (Poncelet et al., 2009). For example, the donor might be genetically modified to protect its cells from the human immune system (Li et al., 2012). MSCs are both immunosuppressive and immunoprivileged and, as such, may be used as an allogeneic source of cells. A recent randomized controlled trial showed significant benefits and low rates of immunologic reactions for transendocardial injection of allogenic BMSCs in patients with ischemic cardiomyopathy (Hare et al., 2012). Another limitation is the time required to produce the MSCs. Cell storage may be used to provide enough material at the time of graft. In a beagle dog, cryopreservation of BMSCs did not alter the periodontal regeneration compared to the use of non-stored cells (Li et al., 2009). However, additional studies are required to ensure the safety of allogenic grafts and cell cryopreservation.

### Adverse effects

With population doublings during the expansion stage, MSCs may be subject to senescence and genetic instability. Progressive shortening of telomeres, modified telomeric structures, and activation of the retinoblastoma protein (pRB) or p53 pathways have been demonstrated to be important triggers for replicative senescence (Schellenberg et al., 2011). Basically, the role of MSCs in cancer can be divided into indirect involvement via the tumor modulatory effect and direct involvement via malignant transformation of themselves (Wong, 2011). The “homing” effect allows MSCs to migrate toward tumor cells, interact with the stroma, and enhance its growth (Wong, 2011). Also, it can lead to malignant transformation of MSCs at the tumor site. While the immunosuppressive properties of MSCs are a good tool for immune disorders, a suppressed immune system may encourage tumor growth in patients with cancer (Shinagawa et al., 2010). To date, no malignant formation has been reported during the course of clinical studies involving periodontal cell therapies (Giordano et al., 2007; Yoshida et al., 2012). Moreover, PDLSC did not induce tumorigenesis after injection into immunodeficient mice for at least 12 weeks (Washio et al., 2010). However, improvements in the understanding of genetic and epigenetic changes will enable researchers to address the risk of tumorigenesis in the context of each type of cell transplantation therapy.

### Cell culture during laboratory phases

The definition of MSCs as advanced-therapy medicinal products in European regulations and the US Food and Drug Administration requirements implies the use of production processes in accordance with Good Manufacturing Practices (GMPs). Requirements concern the environment, staff training and qualification, and controls (Sensebe et al., 2013). Culture conditions are not sufficiently developed to mimic the *in-vivo* cell microenvironment and to ensure that cell proliferation and differentiation can be performed safely (van der Sanden et al., 2010).

Cell culture status, such as culture medium composition and oxygen supply, may interact with MSC expansion and *ex-vivo* properties. Even though they are extensively described in studies, there is no consensus about the required culture conditions. Nevertheless, fetal calf serum (FCS), often used as a supplement, contains a xenogeneic source of growth factors and may transmit animal pathogens (Bourin et al., 2010). Addition of human blood-derived products to replace FCS is secured by serologic and nuclear acid testing for blood-transmitted viruses. Human platelet lysate, a blood plasma enriched in platelet growth factors released by freezing-thawing cycles, may be used to produce clinical-grade, stem-cell-loaded biomaterials as an appropriate FCS substitute in line with clinically applicable practice (Warnke et al., 2013). Various other serum-free media based on mixtures of defined growth factors are also able to maintain the main phenotypic and functional characteristics of cultured MSCs (Chase et al., 2010) but are restricted to research purposes and require upgrading for clinical uses.

### Use of carrier and growth factors

In the review of the literature on periodontal regeneration by MSCs, one of the most striking elements is the numerous carriers that have been used. Cortical bone particulates (Simsek et al., 2012), bovine bone (Fawzy El-Sayed et al., 2012), HA (Ding et al., 2010), polymers (Park et al., 2012), collagen (Suaid et al., 2011), hydrogel (Wei et al., 2010), gelatin (Yang et al., 2010), fibrinogen/thrombin (Hynes et al., 2013), PRP (Tobita et al., 2013), cells as sheets (Tsumanuma et al., 2011), alone or in combination, have been suggested to help cell grafting. The heterogeneity among study methodologies makes it difficult to compare them and to draw sound conclusions. Biomolecules, in particular growth factors, have been suggested for addition to scaffolds to enhance periodontal regeneration (Saito et al., 2009). It has been reported that modified MSCs overexpressing growth factors such as BMP-2 (Chen et al., 2008; Chung et al., 2011) or bFGF (Tan et al., 2009) could improve the healing of periodontal defects by maintaining a long-term release of these factors *in situ* (Ramseier et al., 2012).

### Periodontitis models in animals

Non-human primates are similar to humans, with comparable periodontal tissue structures. However, most non-human primates used for research purposes are expensive and difficult to handle, and ethical restrictions apply to their exploitation (Struillou et al., 2010). The beagle is one of the most commonly used due to its size and its cooperative temperament (Haney et al., 1995; Struillou et al., 2010). Minipigs are also an alternative (Fawzy El-Sayed et al., 2012). However, animal models are unable to mimic some fundamental features: the spontaneous emergence of disease (even though some periodontal lesions can occur in aged animals, periodontal defects are mostly mechanically generated), genetic background, and risk factors (aggressive bacterial flora, occlusal overload, tobacco, prosthesis, systemic diseases of host, and donor, etc.). Overall, these features should be investigated when clinical trials are being designed and analyzed.

### Regulatory information

The translation of a cell-based therapy from bench to bedside is challenging, in a regulatory framework involving multiple responsible authorities. Japan, Europe, and the United States have developed quality standards to regulate cell based therapies with Good Clinical Practice and GMP (Yoshida et al., 2012). In the USA, MSCs are considered in the context of “361 Human Cells, Tissues, or Cellular and Tissue-Based Products” (source: www.fda.gov). For European countries, the framework for human trials is fixed by regulation number 1394/2007 on advanced therapy medicinal products, in force since December 2008.

The clinical development plan should start with the submission of a clinical trial authorization application to the competent authority; clinical trials should be designed to demonstrate the safety and efficacy of cells (George, 2011). It can be noted that, in 2006, the WHO stated that all clinical trials should be registered. Moreover, US federal law (Food and Drug Administration) recommends the registration of trials via clinicaltrials.gov to record key elements of study and basic results, and to report adverse events (Califf et al., 2012).

In first-in-man studies, specific safety endpoints may need to be defined to explore: cross-contamination in cases of allogeneic and xenogeneic graft, chromosomal stability, contamination with microorganisms, safety of engineering devices, stemness potential, functional characterization, and cell phenotype (Dittmar et al., 2010). Because of the risk-benefit balance in periodontal regeneration, emphasis should be placed on the safety of these therapeutics.

## Conclusion

Animal studies suggest that mesenchymal stem cells are effective and safe for periodontal regeneration. Nevertheless, additional studies are needed to improve periodontal cell therapy regeneration and to decipher the biological mechanisms that are involved. For example, a recent study in dogs suggested that periodontal regeneration could be obtained with stem-cell conditioned medium, possibly thanks to multiple cytokines (Inukai et al., 2013). To materialize the translation of cell therapy from the laboratory to the dental chair a compromise, providing real benefits for patients, respecting biosafety requirements, available at affordable prices, covered by a social security system and sufficiently attractive to encourage industrial companies to invest in its development, is still required. It is possible that cell therapy will be implemented in clinical practice as a routine technique in the future.

### Conflict of interest statement

The authors declare that the research was conducted in the absence of any commercial or financial relationships that could be construed as a potential conflict of interest.
